# Tumor co-expression of progranulin and sortilin as a prognostic biomarker in breast cancer

**DOI:** 10.1186/s12885-021-07854-0

**Published:** 2021-02-22

**Authors:** Karoline Berger, Sara Rhost, Svanheiður Rafnsdóttir, Éamon Hughes, Ylva Magnusson, Maria Ekholm, Olle Stål, Lisa Rydén, Göran Landberg

**Affiliations:** 1 Department of Laboratory Medicine, Institute of Biomedicine, Sahlgrenska Center for Cancer Research, Sahlgrenska Academy, University of Gothenburg, Box 425, Medicinaregatan 1G, SE-13 90 Gothenburg, Sweden; 2 Present address: Department of Surgery, National University Hospital of Iceland, 13-A Hringbraut, Reykjavik, Iceland; 3 Department of Oncology, Region Jönköping County, Jönköping, Sweden; 4 Department of Clinical and Experimental Medicine, Linköping University, Linköping, Sweden; 5 Department of Clinical Sciences, Division of Oncology and Pathology, Lund University, Lund, Sweden

**Keywords:** Breast cancer, Cancer stem cells, Estrogen receptor, Progranulin, Sortilin, Tamoxifen, Targeted therapy, Prognostic, Predictive, Biomarker

## Abstract

**Background:**

The growth factor progranulin has been implicated in numerous biological processes such as wound healing, inflammation and progressive tumorigenesis. Both progranulin and its receptor sortilin are known to be highly expressed in subgroups of breast cancer and have been associated with various clinical properties including tamoxifen resistance. Recent data further suggest that progranulin, via its receptor sortilin, drives breast cancer stem cell propagation in vitro and increases metastasis formation in an in vivo breast cancer xenograft model. In this retrospective biomarker analysis, we aimed to determine whether tumor co-expression of progranulin and sortilin has prognostic and treatment predictive values for breast cancer patients.

**Methods:**

We explored how co-expression of progranulin and sortilin was associated with established clinical markers by analyzing a tissue microarray including 560 randomized premenopausal breast cancer patients receiving either 2 years of tamoxifen treatment or no adjuvant treatment, with a median follow-up time of 28 years. Breast cancer-specific survival was analyzed using Kaplan-Meier and Cox Proportional Hazards regression models to assess the prognostic and predictive value of progranulin and sortilin in relation to known clinical markers.

**Results:**

Co-expression of progranulin and sortilin was observed in 20% of the breast cancer samples. In untreated patients, prognostic considerations could be detailed separately from treatment prediction and the high progranulin and sortilin expressing subgroup was significantly associated with breast cancer-specific death in multivariable analyses (HR=2.188, CI: 1.317–3.637, *p*=0.003) along with tumor size, high tumor grade and lymph node positivity. When comparing the untreated patients with tamoxifen treated patients in the ERα positive subgroup, co-expression of progranulin and sortilin was not linked to tamoxifen resistance.

**Conclusion:**

Data suggest that co-expression of progranulin and its receptor sortilin is a novel prognostic biomarker combination identifying a highly malignant subgroup of breast cancer. Importantly, this subpopulation could potentially be targeted with anti-sortilin based therapies.

**Supplementary Information:**

The online version contains supplementary material available at 10.1186/s12885-021-07854-0.

## Background

Breast cancer is the most common cancer in women worldwide. Even though early detection rates and existing therapies contribute to a slightly enhanced survival, many patients experience metastasis and tumor relapse. As a result, breast cancer remains the leading cause of cancer death among the female population [1]. Treatment failure and cancer recurrence are multifactorial but most likely influenced by drug resistance and self-renewal properties of the small population of tumor cells termed cancer stem cells (CSCs) [2]. Further, breast cancer is in many aspects a heterogeneous disease, including subtypes with diverse phenotypes and clinical behaviors [3–5]. Each subtype is responsive to different treatment regimes. Patients that express the estrogen receptor alpha (ERα) are treated with endocrine adjuvant therapy, such as tamoxifen or an aromatase inhibitor. However, despite that endocrine therapy improves the survival of the ERα positive patient group, many patients experience tumor relapse or therapy resistance [6]. Consequently, there is an obvious need to further identify key mediators involved in breast cancer progression in order to optimally distinguish subgroups of breast cancer patients that will benefit from specific treatments or having tumors with inherent aggressive properties.

The pleiotropic growth factor progranulin has been identified as a key mediator involved in breast cancer progression and is further influenced by the tumor microenvironment, which can lead to breast CSC propagation and drug resistance [7–12]. We recently observed that progranulin secretion is induced in ERα positive breast cancer cells exposed to a hypoxic environment, which further induced breast CSC propagation [7]. In fact, progranulin present in serum has been demonstrated to predict recurrence in hormone positive (ERα and progesterone receptor (PR) positive) breast cancer patients during tamoxifen treatment [13]. In addition, high levels of progranulin expression in tumors from patients with ERα positive invasive ductal carcinoma (IDC) is associated with increased risk of recurrence [14]. Further, Li and colleagues demonstrated that progranulin tumor expression was significantly higher in tumors from triple negative breast cancer patients without presence of lymph node metastasis [15]. These triple negative breast tumors also expressed high levels of vascular endothelial growth factor and cluster of differentiation 105 (Endoglin) and high progranulin expression further correlated with epidermal growth factor receptor, suggesting that progranulin is involved in the high angiogenesis in this specific subset of breast cancer.

In addition, progranulin is involved in various biological processes, such as wound healing, tumorigenesis, inflammation and has been associated with various neurological diseases [16–23]. The fact that high progranulin has been observed in both tissue and serum of various cancer types compared to normal tissue [9, 12, 13, 24–30] suggests that progranulin may be a relevant biomarker in breast cancer, as well as in other cancer types [13, 14].

Moreover, sortilin is a known progranulin binding receptor [31], highly expressed in breast cancer cell lines compared to non-tumorigenic breast epithelial cells [32]. Sortilin has also been associated with increased metastatic potential in both IDC and invasive lobular carcinoma (ILC) [32], suggesting that the progranulin receptor could also be involved in breast cancer progression.

In this study, we investigated tumor specific expression and potential clinical associations for progranulin and its associated receptor sortilin, with the aim to identify optimal biomarkers for breast cancer progression and prognosis that could potentially be targeted by anti-sortilin based therapy. This study was performed according to the REMARK guidelines (presented in Additional file 1) using tissue microarrays (TMAs) from a randomized tamoxifen trial including 560 premenopausal breast cancer patients.

## Methods

### Ethics statement

The original study (SBII:2) was approved by the Ethics Committee at Lund and Linköping Universities, Sweden (Dnr LU 240–01 and for the continuation of the study: Dnr Linköping 01–134 and Dnr LU 2015–350). Randomization was performed by the Regional Oncological Centers and oral informed consent was registered for all patients. The data were analyzed anonymously.

### Patients and tumor samples

This retrospective study includes an invasive breast cancer cohort consisting of 560 premenopausal patients enrolled in a randomized clinical trial from 1984 to 1991, where patients received either 2 years of tamoxifen treatment (*n*=275) or no systemic treatment (randomized untreated) (*n*=285). Each patient underwent surgery (either radical mastectomy or breast-conserving surgery) followed by radiotherapy, and in a small number of cases adjuvant polychemotherapy (> 2%). All patients were followed-up for breast cancer-specific survival (BCSS) with up to 32 years of follow-up data. BCSS was calculated as the time from surgery of the primary breast tumor to death from breast cancer. The median post-surgery follow-up time without a breast cancer-specific death was 28.41 years. ERα status was determined by immunohistochemistry or enzyme immunoassay, progesterone receptor (PR) status by immunohistochemistry and human epidermal growth factor receptor 2 (HER2) status was determined by in situ hybridization and immunohistochemistry, as previously described and with a cut-off value of 10% to assess the hormone status of ERα, PR and HER2 [33, 34]. Among the 444 patients analyzed for progranulin and sortilin expression, 317 were considered ERα positive and 96 patients were ERα negative; 230 patients were PR positive, whereas 124 were negative for PR. For HER2 status, 358 patients were HER2 negative and 60 patients HER2 positive. A CONSORT diagram for the trial profile is given in Additional file 5: Fig. S1 and clinical and tumor characteristics for the two study groups are presented in Table 1. Additional details of the trial have been described previously [33, 35, 36].

**Table 1:** Clinicopathological characteristics of the breast cancer patient cohort

Parameter	Randomized untreated (***n***=232)	Tamoxifen (***n***=212)	Total (***n***=444)
**Age at diagnosis (year)**			
Median	45.00	45.00	45.00
Range	26–57	25–57	25–57
**Follow-up time without death from breast cancer**			
Median	28.09	28.56	28.41
10th percentile	20.48	21.35	20.56
90th percentile	30.85	30.72	30.74
**Tumor size (mm)**			
Median	23.00	25.00	24.00
Range	2–50	8–75	2–75
**Tumor histology**			
Ductal	194	176	370
Lobular	18	17	35
Medullar	13	9	22
Missing: 17			
**Tumor grade**			
Grade 1	31	23	54
Grade 2	100	86	186
Grade 3	96	92	188
Missing: 16			
**Lymph node (LN) status**			
LN Positive	166	144	310
LN Negative	65	67	132
Missing: 2			
**Estrogen receptor (ERα)**			
ERα positive	173	144	317
ERα negative	47	49	96
Missing: 31			
**Progesterone receptor (PR)**			
PR positive	122	108	230
PR negative	63	61	124
Missing: 90			
**Human epidermal growth factor receptor 2 (HER2)**			
HER2 negative	186	172	358
HER2 positive	35	25	60
Missing: 26			
**Progranulin expression**			
High	66	73	139
Low	149	124	273
Missing: 32			
**Sortilin expression**			
High	116	109	225
Low	107	95	202
Missing: 17			

### Antibodies and immunohistochemistry

Representative tumor areas of formalin-fixed and paraffin-embedded tissue material were collected from 444 of the 560 patients and selected for TMA construction and sectioned, followed by deparaffinization and rehydration as previously described [35]. Progranulin and sortilin expression were determined by immunohistochemistry using an Autostainer LINK 48 and the Envision FLEX+ detection system (DAKO). Deparaffinized sections (4.5 μm) were subjected to antigen retrieval by high pressure cocking and DIVA antigen retrieval pH 6.2, followed by blocking with 3% hydrogen peroxide and incubation with primary antibody against progranulin (polyclonal goat anti-Progranulin, #AF2420, R&D Systems 1:1000) and sortilin (polyclonal rabbit anti-Sortilin, #AB16640, ABCAM 1:1000) at room temperature for 1 h. For signal amplification of the primary rabbit anti-Sortilin antibody, EnVision™ FLEX+ Rabbit linker (SM805, DAKO) was used. A secondary antibody (polyclonal rabbit anti-goat immunoglobulins/HRP, #P0449, DAKO 1:100) was used for the progranulin staining, followed by signal amplification using EnVision™ FLEX+ Rabbit linker (SM805, DAKO). Further, the EnVision FLEX/HRP visualization reagent EnVision™ FLEX/HRP secondary antibody-coated polymer peroxidase complexes (#SM802, DAKO) was used, followed by DAB substrate/chromogen (DAKO). Slides were counterstained with hematoxylin (DAKO) and stained sections were scanned by a Leica SCN400 scanner at 20X. Antibody validation for IHC assessment of sortilin and progranulin has been performed previously by siRNA knockdown, protease degradation and western blot analysis [31, 37–39]. Additionally, antibody validation has been performed in this study, using IHC and western blot analysis, including siRNA knockdown and chemical degradation on established breast cancer cell lines (see Additional files 2, 3, 4).

### Scoring

Evaluation and scoring of progranulin and sortilin tumor expression were performed independently by a pathologists (Landberg) and a trained breast cancer surgeon (Rafnsdóttir) without knowledge of pathological or clinical data. The scoring system was implemented using an Allred scoring system, ranging from 1 (no/low staining), 2 (low/intermediate staining), 3 (intermediate/high staining) to 4 (high staining). Expression of progranulin and sortilin was evaluated in cancer cells only. There was a similar expression profile between the replicates of the same tumor throughout the cohort. For progranulin, 78% of the scores were based on two replicates and 22% of one replicate with no variation between replicates. For sortilin, 76% of the scores were based on two replicates and 24% of one replicate. Here, 85% of the total replicates were similar. In high and low, 93% were similar. For both sortilin and progranulin there were a divergence in the independent scoring judgement of 12%, however, only 0–2% affected the grouping high/low.

### Statistical analysis

All statistical calculations and modelling were performed in SPSS software version 25 (SPSS, Chicago, IL), GraphPad Prism version 7.00 (GraphPad Software, San Diego, CA) or RStudio version 3.6.2 (packages *stats*, *ggplot2*, *rms*, *survival* and *survminer*). Spearman’s rank-order Correlation Coefficient was used to test the significance of the association between progranulin and sortilin scoring. The relationship between progranulin and sortilin scoring and various parameters were analyzed using Pearson’s Chi-square test for categorical variables and Kruskal-Wallis (or Man-Whitney U) test for continuous variables. Kaplan-Meier curves were used to estimate BCSS, and the log-rank test was used to compare BCSS among different staining scores and treatments, as well as to calculate Hazard Ratio (HR) and 95% Confidence Interval (CI) in these groups. Univariate and multivariable analysis were performed using Cox proportional hazard model for relative risk estimation of different variables, including tumor grade, tumor size, age, lymph node status and ERα status, to compare BCSS among different treatment groups. For univariate and multivariable analysis, HR and 95% CI were calculated. Performance of the multivariable models were measured using the concordance index (C-index) and the proportional hazards assumptions were tested by Schoenfeld residuals. A 10-fold cross-validation, repeated 100 times, was performed to validate the cohort and estimate the prediction accuracy of the fitted model. All *p*-values correspond to two-sided tests, and *p*-values of < 0.05 were considered statistically significant.

## Results

### Correlation between progranulin and sortilin expression and clinicopathological parameters

In order to validate the potential prognostic as well as treatment predictive value of progranulin and sortilin tumor expression, we analyzed 444 breast cancer samples arranged in TMAs that were successfully stained for progranulin and sortilin using immunohistochemistry (IHC). Clinicopathological and molecular parameters included in the study are summarized in Table 1. The median age of the patients was 45 years (range 25–57) and the median follow-up period was 28.41 years. At the last follow-up, 206 (46.4%) of the 444 patients analyzed had died of breast cancer. To predict the accuracy of the multivariable model, a 10-fold cross-validation, repeated 100 times, was performed and demonstrated equivalent results (C-index: 0.642 for all patients in the full model and mean C-index of the repeated test-sets: 0.616). For the cohort studied, there was a significantly increased BCSS for tamoxifen treated patients having ERα positive cancer (*p*=0.031, *n*=384) (see Additional file 5: Fig. S2).

**Fig. 1:**
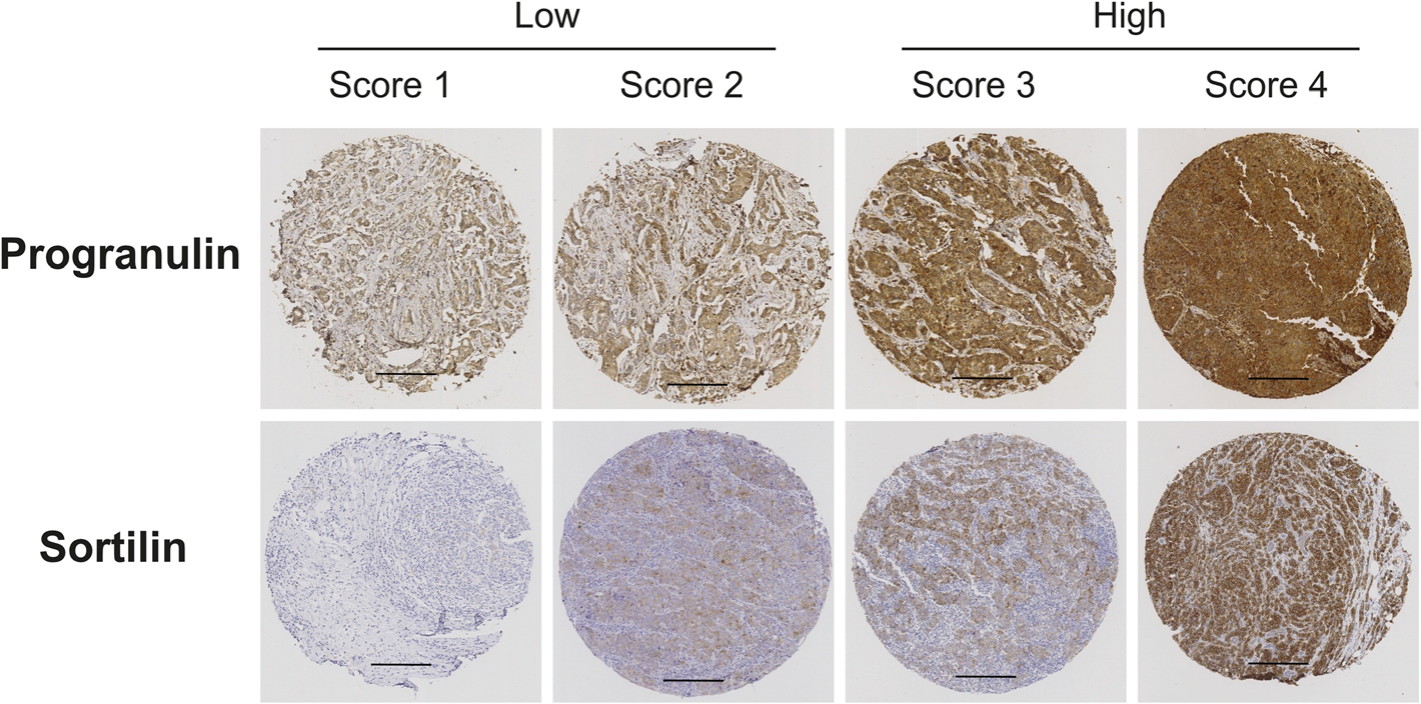
Progranulin and sortilin scoring. Representative immunohistochemical images of breast cancer tissue microarray sections showing variations in progranulin and sortilin expression, scored from 1 to 4, respectively. Brown: positive progranulin/sortilin antibody staining, blue/pale pink: hematoxylin/eosin for nucleus and cytoplasm staining. Scale bar represents 250 μm

Breast cancer-specific progranulin and sortilin protein expression demonstrated very clear staining patterns with cytoplasmic staining without any obvious membrane staining and total lack of nuclear staining, as illustrated in Fig. 1. Progranulin and sortilin protein expression were scored into four groups (illustrated in Fig. 1) and further subdivided into low expression (score 1–2) or high expression (score 3–4). Among the 444 primary breast tumors selected, 412 tumors were successfully stained for progranulin where 273 tumors (66.26%) were categorized as having low progranulin levels (score 1–2) and 139 tumors (33.74%) had high progranulin expression (score 3–4). For sortilin expression analysis, 427 of the 444 breast tumors were successfully stained and 225 tumors had high sortilin expression (score 3–4) (52.69%) and 202 tumors had low expression of sortilin (47.31%) (score 1–2) (Table 1).

Next, we investigated how progranulin and sortilin protein expression were associated with established clinicopathological parameters. In support for a biological association, progranulin and sortilin protein expression correlated significantly (*r*=0.112, *p*=0.026) (see Additional file 5: Table S1). Further, progranulin tumor expression was significantly linked to histological grade (*p*< 0.001), where patients with high-grade tumors showed high progranulin expression, in addition to Ki-67 (*p*=0.001) and the hypoxic marker hypoxia-inducible factor 1-alpha (HIF1α) (*p*=0.002) (see Additional file 5: Table S1). There was also a significant association between progranulin and ERα status (*p*< 0.001), as well as PR status (*p*=0.001) (see Additional file 5: Table S1), where ERα positive tumors tend to have lower progranulin expression, which is consistent with previous findings [19]. In addition, sortilin expression was significantly associated with ERα, where ERα positive tumors tend to have higher sortilin (*p*=0.004) and PR expression (*p*< 0.001). Further, age was also significantly linked to sortilin expression (*p*=0.040) (see Additional file 5: Table S2).

### Patients with high tumor co-expression of progranulin and sortilin had impaired BCSS

Since the aim of this study was to evaluate the aggressiveness of breast cancers expressing both progranulin and the receptor sortilin, the material was subdivided into four groups based on progranulin and sortilin co-expression: 1; low progranulin/low sortilin, 2; low progranulin/high sortilin, 3; high progranulin/low sortilin and 4; high progranulin/high sortilin (see Additional file 2: Table S3). Out of 395 scored tumors, 79 (20%) expressed high levels of both progranulin and sortilin, 56 (14.18%) had high progranulin/low sortilin expression, 129 (32.66%) had high sortilin/low progranulin, and 131 (33.16%) expressed low levels of both markers.

**Fig. 2:**
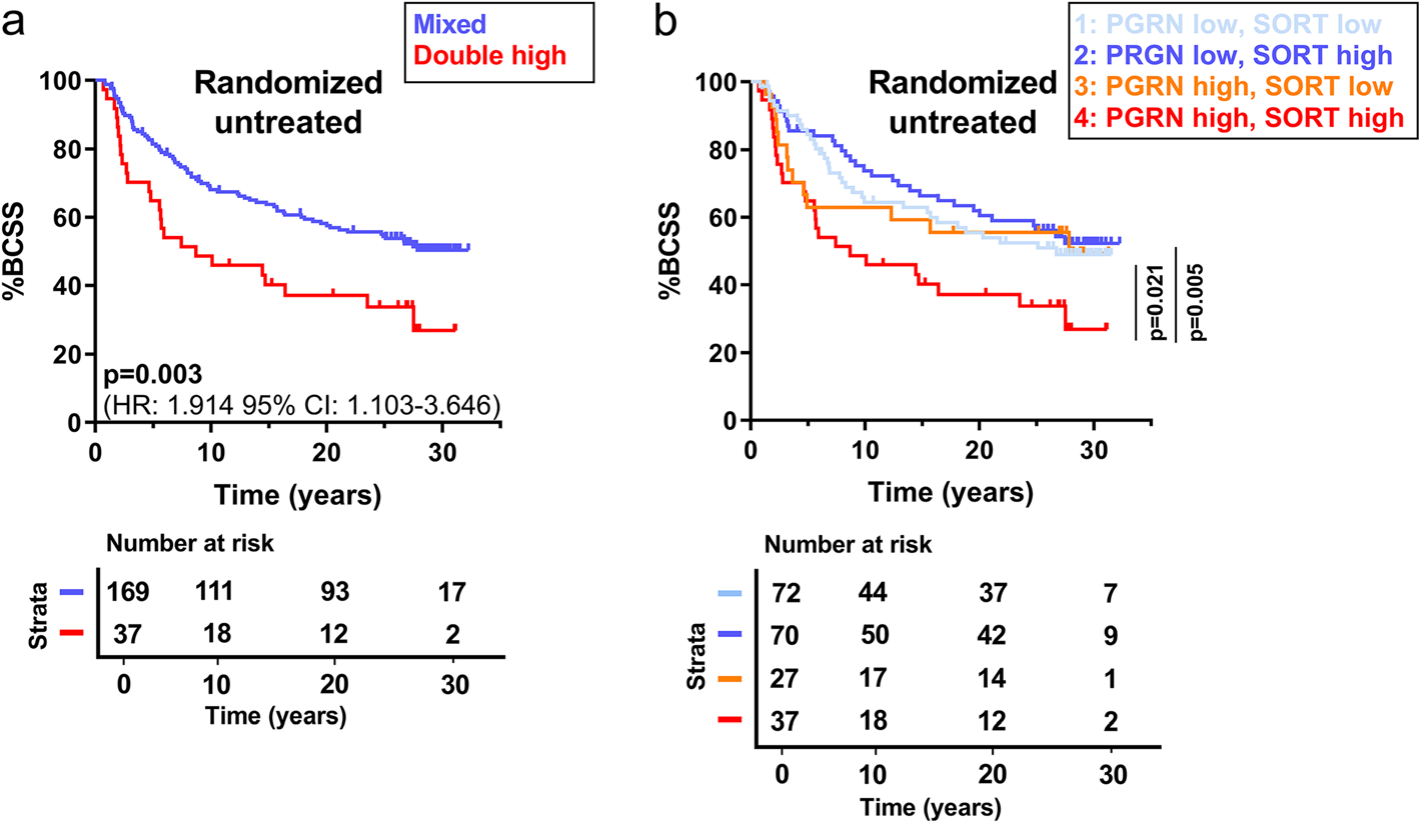
Untreated patients with high tumor co-expression of progranulin and sortilin have worse breast cancer-specific survival. **a** Kaplan-Meier curves illustrating breast cancer-specific survival on combined progranulin and sortilin expression, making a double high group (high progranulin, high sortilin expression) against all other combinations (of low/high progranulin/sortilin expression) for the randomized untreated patients only (*n*=206). **b** BCSS looking at all different progranulin/sortilin combinations in randomized untreated group only (*n*=206). The statistical differences between the curves, as well as HR and 95% CI were estimated by the log-rank test. BCSS: breast cancer-specific survival, HR: hazard ratio, CI: confidence interval, PGRN: progranulin, SORT: sortilin

For the analysis of progranulin and sortilin expression in relation to BCSS, we initially concentrated on the randomized untreated patients to obtain prognostic information not affected by adjuvant tamoxifen treatment (all univariate data Table 2, left). Interestingly, the double high progranulin and sortilin group was significantly different from the remaining subgroups and also associated with worse outcome, as illustrated in Fig. 2a (*p*=0.003, *n*=206). When indicating all four subgroups, the double high subgroup separated significantly from the two subgroups of low progranulin expression (*p*=0.021 and *p*=0.005), whereas there was a non-significant trend for a difference between the double high group and progranulin high group with low sortilin expression (*p*=0.170) (Fig. 2b). In order to clarify the significance of adding sortilin expression to progranulin, we performed multivariable Cox Proportional Hazard (CPH) regression analyses, only analyzing patients with high progranulin tumor expression. In support for an important additive function for sortilin in the progranulin high patient group, high tumor tissue expression of sortilin was significantly linked to BCSS (HR=3.013, 95% CI: 1.219–7.448, *p*=0.017) together with lymph node (LN) positivity (HR=3.854, 95% CI: 1.666–8.919, *p*=0.002) and tumor size (HR=1.089, 95% CI: 1.037–1.143, *p*=0.001) (C-index: 0.701) (see Additional file 5: Table S4).

**Table 2:** Cox regression analysis on randomized untreated patients. Univariate and multivariable interaction analysis on breast cancer-specific survival evaluating various prognostic parameters for relative risk estimation for the untreated patient cohort. Multivariable model adjusted for grade, lymph node status, tumor size, age, ERα and HER2 status, in addition to the progranulin/sortilin scoring combination. HR: hazard ratio, CI: confidence interval, LN: lymph node, ERα: estrogen receptor alpha, HER2: human epidermal growth factor receptor 2

Variable	Univariate analysis			Multivariable analysis		
	HR	95% CI	***p***	HR	95% CI	***p***
**Grade**						
I-II	1			1		
III	1.741	1.210–2.504	**0.003**	1.737	1.054–2.860	**0.030**
**LN status**						
LN negative	1			1		
LN positive	1.964	1.244–3.101	**0.004**	2.250	1.348–3.758	**0.002**
**Tumor size**						
Continuous (mm)	1.014	0.997–1.032	0.108	1.020	0.999–1.041	0.059
**Age**						
Continuous (per year)	0.966	0.936–0.997	**0.033**	0.968	0.934–1.003	0.077
**ERα**						
ERα negative	1			1		
ERα positive	0.824	0.525–1.294	0.401	1.259	0.678–2.340	0.466
**HER2**						
HER2 negative	1			1		
HER2 positive	1.376	0.849–2.230	0.195	1.152	0.648–2.046	0.629
**Progranulin/sortilin combination**						
Mixed	1			1		
Double high progranulin/sortilin	1.922	1.224–3.017	**0.005**	2.188	1.317–3.637	**0.003**

The univariate analysis (Table 2), linking the double high progranulin and sortilin to reduced BCSS in untreated patients, was further validated by multivariable CPH regression analysis in the two subsets of patients including progranulin and sortilin co-expression as well as regular prognostic parameters available for the study. Results showed that high progranulin and sortilin co-expression, together with grade and LN status, were identified as significant risk factors for BCSS (double high: HR=2.188, 95% CI: 1.317–3.637, *p*=0.003, high grade: HR=1.737, 95% CI: 1.054–2.860, *p*=0.030, LN positivity; HR=2.250, 95% CI: 1.348–3.758, *p*=0.002, respectively) (C-index: 0.667) (Table 2, right). Next, we included all patients available within the randomized study in order to increase the statistical power of the data. In this extended patient material, including tamoxifen treated patients, high co-expression of progranulin and sortilin was significantly linked to BCSS (*p*=0.003, *n*=395) as illustrated in Additional file 5: Fig. S3. In addition, univariate and multivariable CPH analysis on all patients revealed comparable results as for the untreated patients (C-index: 0.642) (see Additional file 5: Table S5). Here, endocrine treatment with tamoxifen was also identified as an independent prognostic variable (HR=0.710, 95% CI: 0.517–0.974, *p*=0.034). Interestingly, the double high group was not associated with any of the established clinicopathological parameters, including grade (*p*=0.063) and Ki67 (*p*=0.066) (see Additional file 5: Table S6).

### High tumor co-expression of progranulin and sortilin was not associated with tamoxifen resistance

**Fig. 3:**
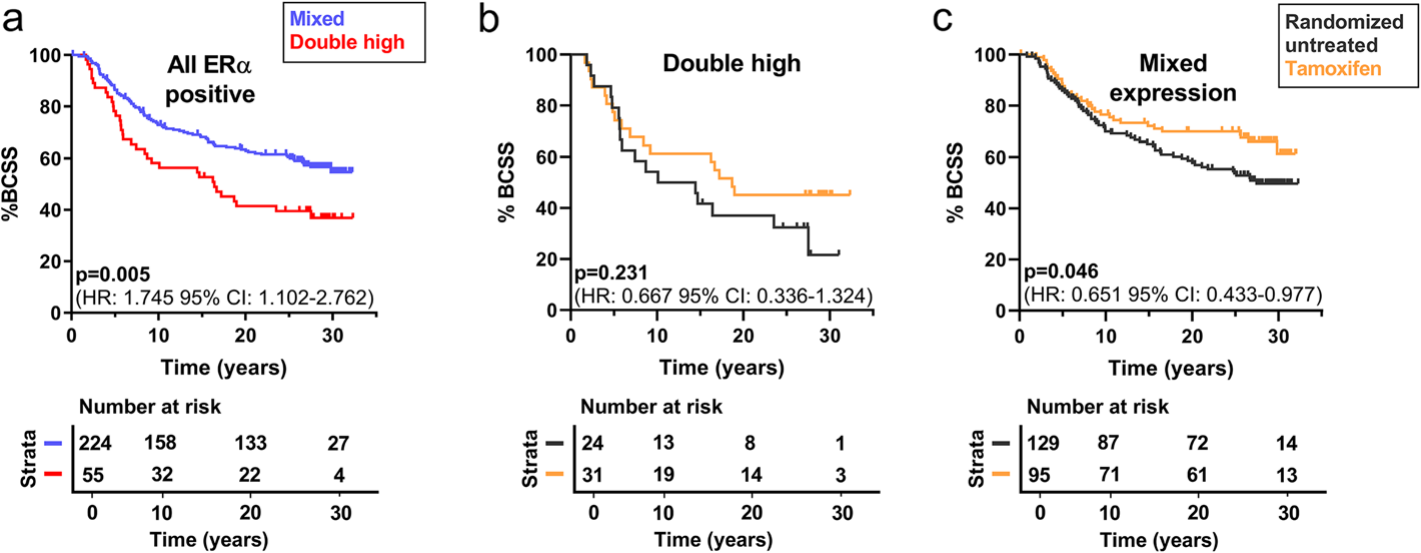
ERα positive patients with high progranulin and sortilin co-expression have worse breast cancer-specific survival. **a**-**c** Kaplan-Meier curves illustrating breast cancer-specific survival in all ERα positive patients. **a** BCSS in the double high group (high progranulin, high sortilin expression) against all other combinations (mixed group) in the ERα positive population (*n*=279). **b**-**c** Tamoxifen response shown by BCSS in all ERα positive patients stratified by **b** double high group (*n*=55) or **c** mixed progranulin/sortilin expression (*n*=224). The statistical differences between the curves, as well as HR and 95% CI were estimated by the log-rank test. BCSS: breast cancer-specific survival, HR: hazard ratio, CI: confidence interval, PGRN: progranulin, SORT: sortilin

Since the analyzed cohort include randomized untreated and tamoxifen treated patients, we could define a potential tamoxifen response or resistance in the subgroup of patients with high co-expression of progranulin and sortilin. These analyses were restricted to patients with ERα positive breast cancer, where high tumor co-expression of progranulin and sortilin demonstrated a significantly worse BCSS compared to mixed groups (*p*=0.005, *n*=279) (Fig. 3a) similar to all samples described above. In addition, we observed that the co-expression of progranulin and sortilin has a significantly worse BCSS compared to patients in the mixed group also in the ERα positive breast cancer patients treated with tamoxifen (*p*=0.034 *n*=126), representing the treatment status of today’s ERα positive breast cancer patients (see Additional file 5: Fig. S4).

Further, in the ERα positive subgroup, multivariable analysis revealed that the double high group (HR=1.980, 95% CI: 1.308–2.996, *p*=0.001) as well as grade (HR=1.612, 95% CI: 1.086–2.394, *p*=0.018) and HER2 positivity (HR=1.716, 95% CI: 1.027–2.867, *p*=0.039) were significantly associated with reduced BCSS in ERα positive breast cancer and that tamoxifen treatment significantly improved ERα positive BCSS (HR=0.628, 95% CI: 0.431–0.915, *p*=0.015) (C-index: 0.637) (see Additional file 5: Table S7). When analyzing the response to tamoxifen treatment, the ERα positive patients with double high expression revealed no significant improvement in BCSS comparing untreated patients with the tamoxifen treated group (*p*=0.231, *n*=55) (Fig. 3b) in contrast to the remaining group with mixed progranulin and sortilin expression (*p*=0.046, *n*=224) (Fig. 3c). This potential difference in tamoxifen response was nevertheless not significant in an interaction analysis (*p*=0.971), suggesting that despite the lack of significant response to tamoxifen treatment in the univariate analysis, the double high group was not resistant to tamoxifen treatment. Altogether, these results suggest that high co-expression of progranulin and sortilin recognizes an ERα positive patient group that could benefit from complementary therapy, possibly targeting sortilin.

## Discussion

Despite valuable traditional cancer therapies, many breast cancer patients experience relapse and therapy resistance. Thus, it is vital to continue to search for mediators driving tumor progression and identify biomarkers that better predict high-risk patients having breast tumors with more aggressive and potentially therapy-resistant behavior. The interest in progranulin has emerged over the last years, with publications demonstrating an overexpression of progranulin in different cancer types and associations with poor prognosis and survival [9, 12, 13, 24–30]. Further, the progranulin receptor sortilin has been linked to breast cancer aggressiveness as well as being expressed in other types of cancer, such as prostate and ovarian cancer [32, 40, 41]. Recent studies from our group have emphasized the stem cell propagating effect by progranulin through its receptor sortilin [7], indicating that this pathway could be central in mediating CSC properties during tumor progression. The existence of a targetable receptor further suggest that future cancer therapies could be developed, selectively targeting CSC propagation via sortilin.

Here, we analyzed the expression levels of both progranulin and its receptor sortilin in a large and unique randomized clinical trial with long-term follow-up in order to clarify if tumor co-expression defined any specific breast cancer type in relation to clinical aggressiveness. The results indeed revealed that high co-expression of progranulin and sortilin could be detected in 20% of the patients and was associated with decreased BCSS. In support for an important function of progranulin and sortilin activation in breast cancer progression, multivariable regression analysis identified high co-expression of progranulin and sortilin, as well as histological grade and lymph node status as independent risk factors.

Previous reports have associated high progranulin expression with ERα negative patients [19] as well as a predictive marker for recurrence in ERα positive breast cancer [14]. We recently showed that progranulin secretion increased in ERα positive breast cancer when cells were subjected to hypoxia, whereas ERα negative breast cancer cells had constitutive high secretion of progranulin [7]. Here we observed a significant link between progranulin and HIF1α, where tumors with high expression of HIF1α tend to express high progranulin. The positive link of progranulin expression with HIF1α suggests a hypoxic influence on progranulin expression, which is in line with previous published data [7]. Further, we observed that high progranulin expression tend to associate with ERα negative status. In contrast, high sortilin expression was associated with ERα positive tumors, which suggests that even though progranulin associated with sortilin, their respective link to ERα are different. Although, the clinical relevance of hypoxic driven progranulin induced CSC propagation in different breast cancer subtypes needs to be studied further.

Sortilin has previously been associated with breast cancer aggressiveness and contributes to tumor cell adhesion and invasion [32]. We recently published that a small molecule inhibitor of sortilin (AF38469) block progranulin induced breast cancer progression in vivo [7, 42]. In this study, orally administration of AF38469 significantly reduced the development of metastasis, which suggest that sortilin may function as a therapeutic target in breast cancer.

Here, in this cohort, high sortilin tumor expression on its own demonstrated no significant reduction of BCSS in either all patients or in the randomized control group (a Kaplan-Meier plot of the sortilin expression is shown in Additional file 6). However, the combination of high tumor co-expression of progranulin and sortilin demonstrated a significantly worse BCSS. Importantly, multivariable analysis revealed that when analyzing the progranulin high subgroup separately, high sortilin expression was identified as a significant prognostic variate linked to worse BCSS together with lymph node positivity and tumor size. This support the fact that sortilin adds prognostic information when combined with progranulin.

Current treatment for patients with ERα positive tumors includes endocrine therapy, such as tamoxifen. Therefore, we additionally analyzed the prognostic value of tumor co-expression of progranulin and sortilin in ERα positive tumors subjected to tamoxifen treatment in order to represent today’s population of luminal breast cancer. Importantly, in this tamoxifen treated ERα positive patient group, the tumor co-expression of progranulin and sortilin also showed a significantly worse BCSS.

Further, previous report described progranulin to be associated with resistance towards tamoxifen therapy [8]. Moreover, another report suggested that progranulin levels predicted recurrence in patients with hormone receptor positive breast cancer during tamoxifen treatment [13]. Here, multivariable interaction analysis identified that the double progranulin/sortilin high ERα positive group was not resistant to tamoxifen treatment, even though univariate analysis demonstrated no significant improvement in BCSS in the tamoxifen treated group. Notably, limitations in this study includes that the tamoxifen treatment in this cohort is restricted to premenopausal women with only 2 years of adjuvant treatment and BCSS may also have been affected by later therapies, which are not considered in this cohort, at disease recurrence.

## Conclusion

In conclusion, we have shown that a combination of high progranulin and high sortilin tumor tissue expression defines a novel and highly malignant subgroup of breast cancer patients. Whether these patients may benefit from complementary targeted anti-sortilin based therapies needs to be investigated.

## Supplementary Information

**Additional file 1:.** REMARK guidelines. Checklist with REporting recommendations for tumor MARKer prognostic studies

**Additional file 2:.** Progranulin antibody validation. Validation of the progranulin antibody (AF2420, R&D Systems) using Western blotting and immunohistochemistry. Weak or no staining was seen in MCF10a and MDA-MB-468, while strong positive staining was seen in MDA-MB-231 and T47D (A). Correspondingly, protein extracts from MDA-MB-231 and T47D gave an intense band at between 70 and 100 kDa using Western blotting and protein extracts from MCF10a and MDA-MB-468 produced very weak bands (B, left). Knockdown experiments using either Scr. Control or siGRN confirmed progranulin antibody specificity (B, right). Representative images of three independent experiments. Scale bar represents 100 μm.

**Additional file 3:.** Sortilin antibody validation. Validation of the sortilin antibody (ab16640, Abcam) using Western blotting and immunohistochemistry. Weak or no staining was seen in MCF10a and CAL-120, while strong positive staining was seen in T47D and MCF7 (A). Correspondingly, protein extracts from T47D and MCF7 gave an intense band at ~ 95 kDa using Western blotting and protein extracts from MCF10a and CAL-120 produced only faint bands (B). Knockdown experiments using either (C) Scr. Control or siSORT1, as well as (D) treating cells with a sortilin degrader, MPEP (M; 1–1[2-(2-tert-butyl-5-methylphenoxy)-ethyl-3-methylpiperidine; Lee, Almeida et al. 2014) confirmed sortilin antibody specificity in T47D. Representative images of three independent experiments. Scale bar represents 100 μm.

**Additional file 4: **GRN and SORT1 gene expression in various cell lines. (A) mRNA expression of progranulin (GRN) and sortilin (SORT1) were analyzed by qPCR. Primers used were as follows: 5′-CCAAAGATCAGGTAACAACTCCG-3′ (forward strand) and 5’CATCGACCATAACACAGCACG − 3′ (reverse strand) for GRN and 5′-ATGGGAAGAAATCCACAAAGCAG − 3′ (forward strand) and 5′-ATTCCAGAGCCCCAAGGTCAG-3′ (reverse strand) for SORT1 and 5′- GATGCGTGCCCAAGGAC − 3′ (forward strand) and 5′-CAGGTCTAAATCGGGGTGG-3′ (reverse strand) for gene ribosomal protein S26 (RPS26). The results were analyzed using GenEx Software (GenEx 7.0, MultiD Analysis AB) and normalized to those of the housekeeping gene RPS26 (reference gene). Result are shown as mean ±SEM from at least three independent experiments. Statistical significance was calculated using one-way ANOVA adjusted for multiple comparison, where *P< 0.05, **P< 0.01 and ***P< 0.001. (B) Transcriptional profiling performed by Neve and colleagues that the gene expression of GRN and SORT1 is similar to what we have detected at both mRNA and protein level for the relevant cell lines. Data modified from (Neve RM et al. *Cancer Cell.* 2006).

**Additional file 5: Supplementary material. Fig. S1**: CONSORT diagram for the study. Flowchart of the study showing the enrollment of the patients, treatment allocation and analysis. **Fig. S2**: ERα positive patients stratified by treatment arm. Kaplan-Meier estimates showing breast cancer-specific survival in ERα positive breast cancer patients treated with tamoxifen or randomized untreated. **Fig. S3**: Patients with high tumor co-expression of progranulin and sortilin have worse breast cancer-specific survival. Kaplan-Meier curves illustrating breast cancer-specific survival on combined progranulin and sortilin expression, showing high expression of both markers against all other combinations for all patients. **Fig. S4**: Patients with high tumor co-expression of progranulin and sortilin have worse breast cancer-specific survival in the ERα positive patient group treated with tamoxifen. Kaplan-Meier estimates showing breast cancer-specific survival on combined progranulin and sortilin expression, showing high expression of both markers against all other combinations in ERα positive breast cancer patients treated with tamoxifen. **Table S1**: Distribution of progranulin scores according to clinicopathological parameters in the cohort. Statistics on progranulin scoring in relation to clinical parameters. **Table S2**: Distribution of sortilin expression according to clinicopathological parameters in the cohort. Statistics on sortilin scoring in relation to clinical parameters. **Table S3**: Cross table. The relationship between progranulin and sortilin expression in the patient cohort. **Table S4**: Cox regression analysis on randomized untreated patients with high progranulin tumor tissue expression. Multivariable interaction analysis on breast cancer-specific survival evaluating various prognostic parameters for relative risk estimation for the untreated patient cohort having high tumor expression of progranulin. **Table S5**: Cox regression analysis on all patients. Univariate and multivariable interaction analysis on breast cancer-specific survival evaluating various prognostic parameters for relative risk estimation for all patients in the cohort. **Table S6**: Distribution of co-expression of progranulin and sortilin according to clinicopathological parameters in the cohort. Statistics on co-expression of progranulin and sortilin scoring in relation to clinical parameters. **Table S7**: Cox regression analysis on ERα positive patients. Multivariable regression analysis on breast cancer-specific survival evaluating various prognostic parameters for relative risk estimation for the ERα positive patient cohort.

**Additional file 6.** Sortilin tumor expression on its own shows no difference in survival. Kaplan-Meier curves illustrating breast cancer-specific survival according to high or low sortilin expression.

## Data Availability

The dataset analyzed during the current study are available from the corresponding author on reasonable request.

## Abbreviations

BCSS: Breast cancer-specific survival
C-index: Concordance index
CI: Confidence interval
CPH: Cox proportional hazard
CSC: Cancer stem cell
ERα: Estrogen receptor alpha.
HER2: Human epidermal growth factor receptor 2
HIF1α: Hypoxia-inducible factor 1-alpha
IDC: Invasive ductal carcinoma
IHC: Immunohistochemistry
ILC: Invasive lobular carcinoma.
LN: Lymph node
PR: Progesterone receptor
TMA: Tissue microarray
