# Supplementary material for: Tumor co-expression of progranulin and sortilin as a prognostic biomarker in breast cancer
Source: BMC Cancer. 2021 Feb 22;21:185. doi: 10.1186/s12885-021-07854-0 (PMC7898426; doi:10.1186/s12885-021-07854-0)
Supplement: Supplementary file 1 — Additional file 1:. REMARK guidelines. Checklist with REporting recommendations for tumor MARKer prognostic studies [file 12885_2021_7854_MOESM1_ESM.pdf]

The REMARK checklist

	Item to be reported	Page no.
**INTRODUCTION**		
1	*State the marker examined, the study objectives, and any pre-specified hypotheses.*<br>Study objectives: To determine whether tumor co-expression of progranulin and sortilin has prognostic and treatment predictive values for breast cancer patients.<br>Hypothesis: As both progranulin and its functional receptor sortilin are known to be highly expressed in subgroups of breast cancer and have been associated with various clinical properties, including tamoxifen resistance, we hypothesize that cancer specific co-expression of progranulin and sortilin could define a highly malignant subgroup of breast cancer.<br>Examined markers: Antibodies for progranulin (#AF2420, R&D Systems) and sortilin (#AB16640, ABCAM) assessed by immunohistochemistry, in addition to the established markers: age, tumor size, tumor grade, tumor histology, lymph node (LN) status, HER2, progesterone receptor (PR), estrogen receptor (ER) status.	2, 5
**MATERIALS AND METHODS**		
Patients		
2	*Describe the characteristics (e.g., disease stage or co-morbidities) of the study patients, including their source and inclusion and exclusion criteria.*<br>Characteristics of the study participants are detailed in "Patients and tumor samples" in the Methods section, as well as Table 1 and Additional File 5: Figure S1. 564 premenopausal stage II invasive breast cancer patients were enrolled in a randomized trial (SBII:2a) from 1986 to 1991, and selected for two years of tamoxifen treatment (n=276) or no systemic treatment (n=288). Patients were considered premenopausal until one year after last menstruation.	5-7
3	*Describe treatments received and how chosen (e.g., randomized or rule-based).*<br>Patients were randomly assigned and received either two years of adjuvant tamoxifen treatment or no systemic treatment. A daily dosage of 20mg or 40mg of tamoxifen was given. Each patient underwent surgery (either radical mastectomy or breast-conserving surgery with axillary lymph node dissection). The surgery was followed by radiotherapy (50Gy) given to the breast, and in cases where the patients had axillary lymph node metastasis, locoregional radiotherapy were given. In addition, less than 2% of the patients received adjuvant polychemotherapy.	
Specimen characteristics		
4	*Describe type of biological material used (including control samples) and methods of preservation and storage.*<br>Formalin-fixed paraffin-embedded breast tumor tissue of the primary tumor were used for construction of tissue microarrays (TMAs). The samples were stained for antibodies against progranulin (#AF2420, R&D Systems) and sortilin (#AB16640, ABCAM). Random control samples from invasive breast cancer were included in the TMAs and stained with different dilutions and protocols for antigen retrieval before staining the actual TMAs. The protocol that gave specific staining of the tumor cells, but also resulted in samples with negative staining was used for staining the cohort TMAs. The TMAs were stored at room temperature.	6-7
Assay methods		
5	*Specify the assay method used and provide (or reference) a detailed protocol, including specific reagents or kits used, quality control procedures, reproducibility assessments, quantitation methods, and scoring and reporting protocols. Specify whether and how assays were performed blinded to the study endpoint.*<br>Details of the protocols are described in the "Immunohistochemistry" and "Scoring" section under Methods. Antibody specificity was validated using immunohistochemistry on formalin- fixed paraffin-embedded breast cancer cell lines with high or low expression of progranulin and sortilin (positive and negative controls), in parallel to Western blotting of the cell lines in addition to using siRNA and by chemical degradation (see Additional file 2 and 3). Scoring was performed independently without knowledge of pathological or clinical data. There was a similar expression profile between replicates of the same tumor throughout the cohort. For both progranulin and sortilin there were a divergence in the independent scoring judgement of 12%, however, only 0-2% affected the grouping high/low.	6-7
Study design		
6	*State the method of case selection, including whether prospective or retrospective and whether stratification or matching (e.g., by stage of disease or age) was used. Specify the time period from which cases were taken, the end of the follow-up period, and the median follow-up time.*<br>A randomized controlled trial where patients were enrolled in the study between 1986 to 1991. The study presented here was a translational study where the staining and scoring of progranulin and sortilin was performed retrospectively. The median follow-up time without a breast cancer death was 28.41 years. Patient records were re-evaluated in 2016, giving a follow-up time of up to 32 years.	5-6

7	*Precisely define all clinical endpoints examined.*<br>The aim of the study was to compare the effect of tamoxifen treatment and no adjuvant treatment in relation to recurrence-free survival (RFS). RFS included local, regional, distant recurrences and breast cancer-specific death, but not contralateral breast cancer, as the primary event. In this retrospective study we look at the association of high tumor co-expression of progranulin and sortilin in relation to breast cancer specific survival (BCSS). BCSS was calculated as the time from surgery of primary breast cancer to death from breast cancer.	
8	*List all candidate variables initially examined or considered for inclusion in models.*<br>Variables included in the multivariable models include tumor grade, LN status, tumor size, age, treatment, ER and PR status, HER2 status, as well as progranulin and sortilin tumor expression.	
9	*Give rationale for sample size; if the study was designed to detect a specified effect size, give the target power and effect size.*<br>The study was designed and planned to include at least 500 patients in a two-armed study aiming at a 15% difference in outcome between the treatment arms, with 90% power and an alpha level of 5%.	
Statistical analysis methods		
10	*Specify all statistical methods, including details of any variable selection procedures and other model-building issues, how model assumptions were verified, and how missing data were handled.*<br>Statistical methods are specified in the Methods section. Patients with a missing value for one of the variables were excluded from the multivariable analysis. The proportional hazards assumption of the Cox regression was checked using the dedicated functions of the R-packages *survival* and s*urvminer* confirming the assumption of proportional hazards. For internal validation of the multivariable models, a 10-fold cross-validation was performed, repeated 100 times and the C-index was applied to each sample as well as a mean C-index score for the test sets.	7-8
11	*Clarify how marker values were handled in the analyses; if relevant, describe methods used for cutpoint determination.*<br>Details of how marker values were handled and cutpoints determined are provided in the “Immunohistochemistry” and “Scoring” section under Methods, as well as in the Result section and Figure 1. Tumors with score 1-2 were considered having low progranulin (or sortilin) expression and tumors with score 3-4 as having high expression of progranulin (or sortilin).	
**RESULTS**		
Data		
12	*Describe the flow of patients through the study, including the number of patients included in each stage of the analysis (a diagram may be helpful) and reasons for dropout. Specifically, both overall and for each subgroup extensively examined report the numbers of patients and the number of events.*<br>Flowchart of patients and number of events are shown in detail in Additional File 5: Figure S1.	5-8
13	*Report distributions of basic demographic characteristics (at least age and sex), standard (disease-specific) prognostic variables, and tumor marker, including numbers of missing values.*<br>See Table 1.	
Analysis and presentation		
14	*Show the relation of the marker to standard prognostic variables.*<br>See Table 1, Additional File 5: Table S1 and Table S2.	8-12
15	*Present univariable analyses showing the relation between the marker and outcome, with the estimated effect (e.g., hazard ratio and survival probability). Preferably provide similar analyses for all other variables being analyzed. For the effect of a tumor marker on a time-to-event outcome, a Kaplan-Meier plot is recommended.*<br>For all univariate analysis, see Figure 2, Figure 3, Table 2, Additional File 5: Figure S2, Figure S3 and Table S5.	
16	*For key multivariable analyses, report estimated effects (e.g., hazard ratio) with confidence intervals for the marker and, at least for the final model, all other variables in the model.*<br>All multivariable analysis are shown in Table 2, Additional File 5: Table S4, Table S5 and Table S6. Variables were entered into the model in one single step and include tumor grade, LN status, age, tumor size, randomization, ER, HER2 status and double high co-expression of progranulin and sortilin.	
17	*Among reported results, provide estimated effects with confidence intervals from an analysis in which the marker and standard prognostic variables are included, regardless of their statistical significance.*<br>See Figure 2, Figure 3, Table 2, Additional File 5: Figure S2, Figure S3, Table S4, Table S5 and Table S6.	
18	*If done, report results of further investigations, such as checking assumptions, sensitivity analyses, and internal validation.*	

Source: McShane LM, Altman DG, Sauerbrei W, Taube SE, Gion M, Clark GM: Reporting recommendations for tumor marker prognostic studies (REMARK). *J Natl Cancer Inst* 2005; 97: 1180-1184.

	Reported in the Methods section, as well as the Result section. The proportional hazards assumption of the Cox regression was checked using the dedicated functions of the R-packages *survival* and s*urvminer* confirming the assumption of proportional hazards. For internal validation of the multivariable models a 10-fold cross-validation were performed, repeated 100 times and the C-index was applied to each sample as well as a mean C-index score for the test sets.	
**DISCUSSION**		
19	*Interpret the results in the context of the pre-specified hypotheses and other relevant studies; include a discussion of limitations of the study.*<br>The study results were discussed in relation to our pre-specified hypotheses and other relevant studies in the Discussion section. To our knowledge, our results are the first from a randomized trial to show that high tumor co-expression of progranulin and sortilin can be used as a prognostic marker for BCSS (to predict survival).<br>The study has some limitations. Insufficient tumor material (only 444 out of 560 could be stained for progranulin and/or sortilin) lead to a loss of patients. In addition, patients were treated with tamoxifen for two years, not five, due to the advantage of five years of treatment had not yet been established when the study was conducted. No information on subsequent therapies implemented after disease recurrence is taken into account.	12-15
20	*Discuss implications for future research and clinical value.*<br>Deliberated in the Discussion section of the paper. Identification of a highly malignant subgroup of breast cancer in need of additional treatment, potentially involving novel therapy approaches targeting the receptor sortilin.	

Source: McShane LM, Altman DG, Sauerbrei W, Taube SE, Gion M, Clark GM: Reporting recommendations for tumor marker prognostic studies (REMARK). *J Natl Cancer Inst* 2005; 97: 1180-1184.
