# Supplementary material for: Tumor co-expression of progranulin and sortilin as a prognostic biomarker in breast cancer
Source: BMC Cancer. 2021 Feb 22;21:185. doi: 10.1186/s12885-021-07854-0 (PMC7898426; doi:10.1186/s12885-021-07854-0)
Supplement: Supplementary file 2 — Additional file 2:. Progranulin antibody validation. Validation of the progranulin antibody (AF2420, R&D Systems) using Western blotting and immunohistochemistry. Weak or no staining was seen in MCF10a and MDA-MB-468, while strong positive staining was seen in MDA-MB-231 and T47D (A). Correspondingly, protein extracts from MDA-MB-231 and T47D gave an intense band at between 70 and 100 kDa using Western blotting and protein extracts from MCF10a and MDA-MB-468 produced very weak bands (B, left). Knockdown experiments using either Scr. Control or siGRN confirmed progranulin antibody specificity (B, right). Representative images of three independent experiments. Scale bar represents 100 μm. [file 12885_2021_7854_MOESM2_ESM.pdf]

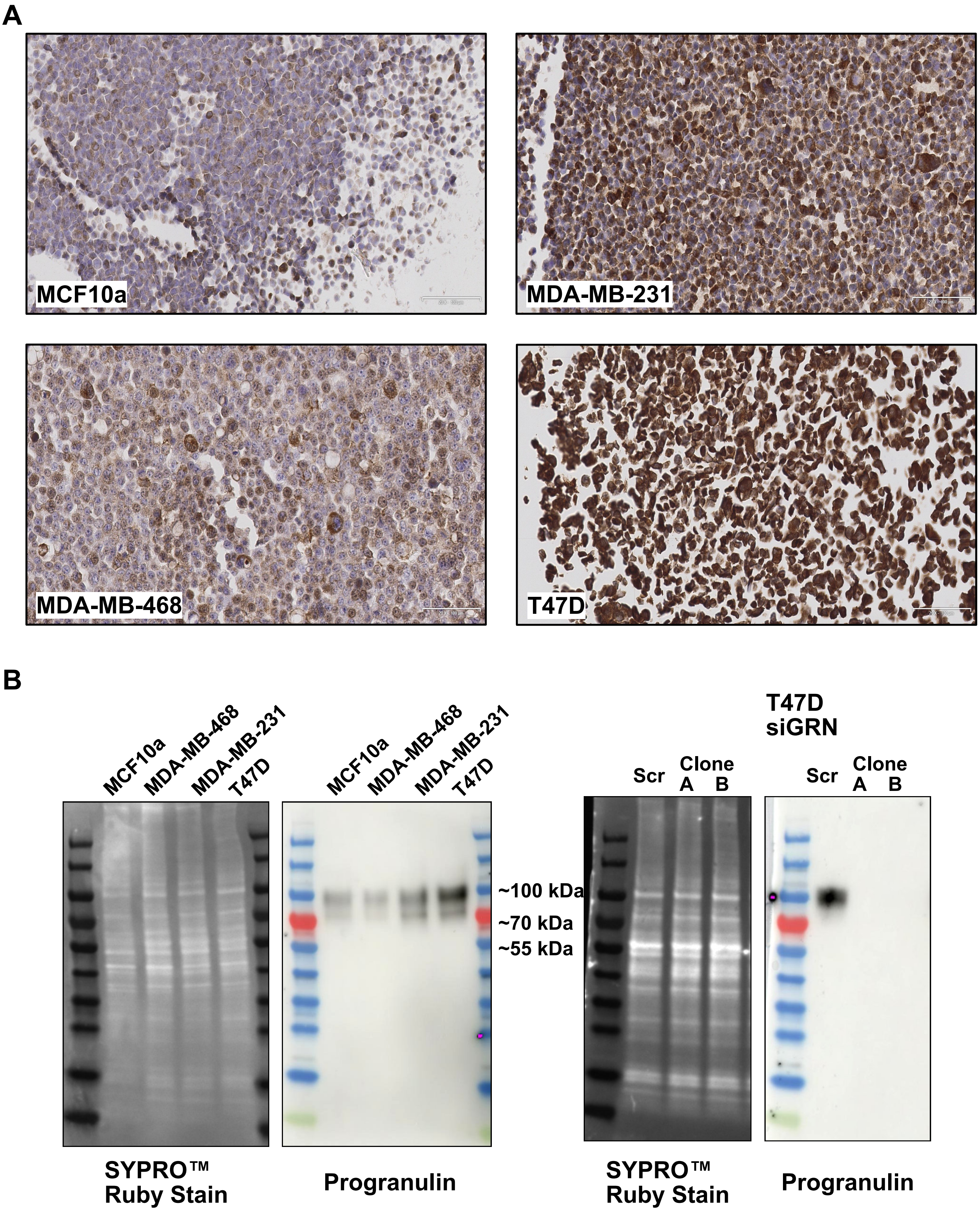

Additional file 2: Progranulin antibody validation.
Validation of the progranulin antibody (AF2420, R&D Systems) using Western blotting and immunohistochemistry. Weak or no staining was seen in MCF10a and MDA-MB-468, while strong positive staining was seen in MDA-MB-231 and T47D (A). Correspondingly, protein extracts from MDA-MB-231 and T47D gave an intense band between 70-100kDa using Western blotting and protein extracts from MCF10a and MDA-MB-468 produced very weak bands (B, left). Knockdown experiments using either Scr. control or siGRN confirmed progranulin antibody specificity in T47D (B, right). Representative images of three independent experiments. Scalebar represents 100µm.
