# Supplementary material for: Tumor co-expression of progranulin and sortilin as a prognostic biomarker in breast cancer
Source: BMC Cancer. 2021 Feb 22;21:185. doi: 10.1186/s12885-021-07854-0 (PMC7898426; doi:10.1186/s12885-021-07854-0)
Supplement: Supplementary file 3 — Additional file 3:. Sortilin antibody validation. Validation of the sortilin antibody (ab16640, Abcam) using Western blotting and immunohistochemistry. Weak or no staining was seen in MCF10a and CAL-120, while strong positive staining was seen in T47D and MCF7 (A). Correspondingly, protein extracts from T47D and MCF7 gave an intense band at ~ 95 kDa using Western blotting and protein extracts from MCF10a and CAL-120 produced only faint bands (B). Knockdown experiments using either (C) Scr. Control or siSORT1, as well as (D) treating cells with a sortilin degrader, MPEP (M; 1–1[2-(2-tert-butyl-5-methylphenoxy)-ethyl-3-methylpiperidine; Lee, Almeida et al. 2014) confirmed sortilin antibody specificity in T47D. Representative images of three independent experiments. Scale bar represents 100 μm. [file 12885_2021_7854_MOESM3_ESM.pdf]

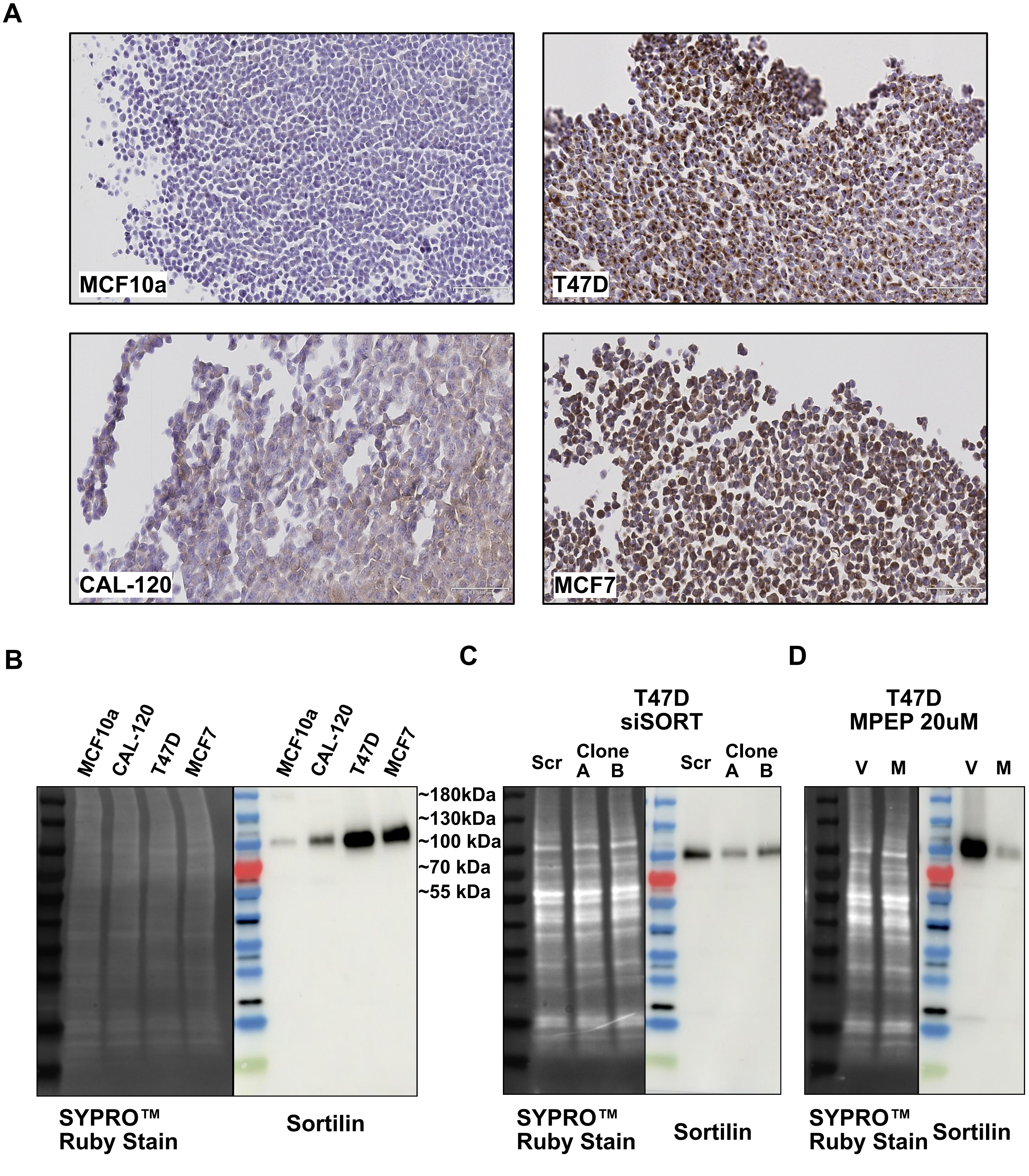

Additional file 3: Sortilin antibody validation.

Validation of the sortilin antibody (ab16640, Abcam) using Western blotting and immunohistochemistry. Weak or no staining was seen in MCF10a and CAL-120, while strong positive staining was seen in T47D and MCF7 (A). Corespondingly, protein extracts from T47D and MCF7 gave an intense band at ~95kDa using Western blotting and protein extracts from MCF10a and CAL-120 produced only faint bands (B). Knockdown experiments using either (C) Scr. control or siSORT, as well as (D) treating cells with a sortilin degrader, MPEP (M; 1-[2-(2-tert-butyl-5-methylphenoxy)-ethyl]-3-methylpiperidine; Lee, Almeida *et al* 2014) confirmed sortilin antibody specificity in T47D. Representative images of three independent experiments. Scalebar represents 100μm.
