# Supplementary material for: Tumor co-expression of progranulin and sortilin as a prognostic biomarker in breast cancer
Source: BMC Cancer. 2021 Feb 22;21:185. doi: 10.1186/s12885-021-07854-0 (PMC7898426; doi:10.1186/s12885-021-07854-0)
Supplement: Supplementary file 4 — Additional file 4: GRN and SORT1 gene expression in various cell lines. (A) mRNA expression of progranulin (GRN) and sortilin (SORT1) were analyzed by qPCR. Primers used were as follows: 5′-CCAAAGATCAGGTAACAACTCCG-3′ (forward strand) and 5’CATCGACCATAACACAGCACG − 3′ (reverse strand) for GRN and 5′-ATGGGAAGAAATCCACAAAGCAG − 3′ (forward strand) and 5′-ATTCCAGAGCCCCAAGGTCAG-3′ (reverse strand) for SORT1 and 5′- GATGCGTGCCCAAGGAC − 3′ (forward strand) and 5′-CAGGTCTAAATCGGGGTGG-3′ (reverse strand) for gene ribosomal protein S26 (RPS26). The results were analyzed using GenEx Software (GenEx 7.0, MultiD Analysis AB) and normalized to those of the housekeeping gene RPS26 (reference gene). Result are shown as mean ±SEM from at least three independent experiments. Statistical significance was calculated using one-way ANOVA adjusted for multiple comparison, where *P< 0.05, **P< 0.01 and ***P< 0.001. (B) Transcriptional profiling performed by Neve and colleagues that the gene expression of GRN and SORT1 is similar to what we have detected at both mRNA and protein level for the relevant cell lines. Data modified from (Neve RM et al. Cancer Cell. 2006). [file 12885_2021_7854_MOESM4_ESM.pdf]

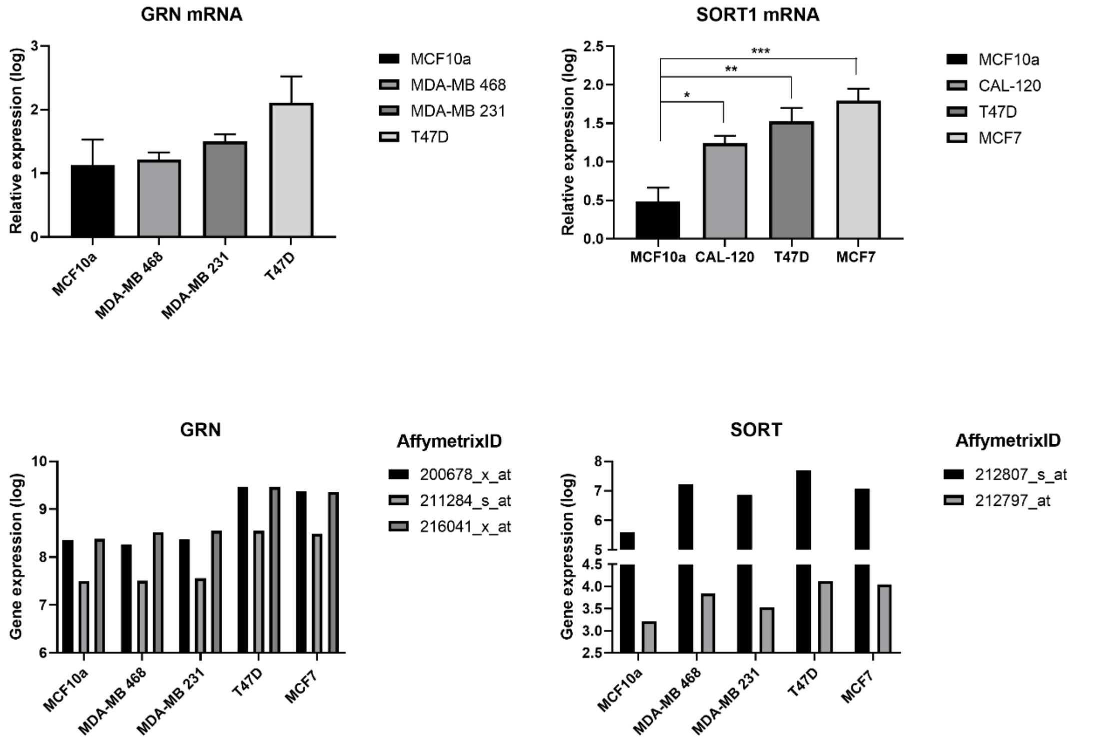

Additional File 4: GRN and SORT1 gene expression in various cell lines.

(A) mRNA expression of progranulin (GRN) and sortilin (SORT1) were analyzed by qPCR. Primers used were as follows: 5'-CCAAAGATCAGGTAACAACTCCG-3' (forward strand) and 5'CATCGACCATAACACAGCACG -3' (reverse strand) for GRN and 5'-ATGGGAAGAAATCCACAAAGCAG -3' (forward strand) and 5'-ATTCCAGAGCCCCAAGGTCAG-3' (reverse strand) for SORT1 and 5'-GATGCGTGCCCAAGGAC -3' (forward strand) and 5'-CAGGTCTAAATCGGGGTGG-3' (reverse strand) for gene ribosomal protein S26 (RPS26). The results were analyzed using GenEx Software (GenEx 7.0, MultiD Analysis AB) and normalized to those of the housekeeping gene RPS26 (reference gene). Result are shown as mean ±SEM from at least three independent experiments. Statistical significance was calculated using one-way ANOVA adjusted for multiple comparison, where **P*<0.05, ***P*<0.01 and ****P*<0.001. **(B)** Transcriptional profiling performed by Neve and colleagues that the gene expression of GRN and SORT1 is similar to what we have detected at both mRNA and protein level for the relevant cell lines. Data modified from (Neve RM *et al. Cancer Cell.* 2006).
