# Supplementary material for: Tumor co-expression of progranulin and sortilin as a prognostic biomarker in breast cancer
Source: BMC Cancer. 2021 Feb 22;21:185. doi: 10.1186/s12885-021-07854-0 (PMC7898426; doi:10.1186/s12885-021-07854-0)
Supplement: Supplementary file 5 — Additional file 5: Supplementary material. Fig. S1: CONSORT diagram for the study. Flowchart of the study showing the enrollment of the patients, treatment allocation and analysis. Fig. S2: ERα positive patients stratified by treatment arm. Kaplan-Meier estimates showing breast cancer-specific survival in ERα positive breast cancer patients treated with tamoxifen or randomized untreated. Fig. S3: Patients with high tumor co-expression of progranulin and sortilin have worse breast cancer-specific survival. Kaplan-Meier curves illustrating breast cancer-specific survival on combined progranulin and sortilin expression, showing high expression of both markers against all other combinations for all patients. Fig. S4: Patients with high tumor co-expression of progranulin and sortilin have worse breast cancer-specific survival in the ERα positive patient group treated with tamoxifen. Kaplan-Meier estimates showing breast cancer-specific survival on combined progranulin and sortilin expression, showing high expression of both markers against all other combinations in ERα positive breast cancer patients treated with tamoxifen. Table S1: Distribution of progranulin scores according to clinicopathological parameters in the cohort. Statistics on progranulin scoring in relation to clinical parameters. Table S2: Distribution of sortilin expression according to clinicopathological parameters in the cohort. Statistics on sortilin scoring in relation to clinical parameters. Table S3: Cross table. The relationship between progranulin and sortilin expression in the patient cohort. Table S4: Cox regression analysis on randomized untreated patients with high progranulin tumor tissue expression. Multivariable interaction analysis on breast cancer-specific survival evaluating various prognostic parameters for relative risk estimation for the untreated patient cohort having high tumor expression of progranulin. Table S5: Cox regression analysis on all patients. Univariate and multivariabl [file 12885_2021_7854_MOESM5_ESM.pdf]

Additional file 5: Supplementary Material

Figure S1: CONSORT diagram for the study.

Figure S2: ERα positive patients stratified by treatment arm.

Figure S3: Patients with high tumor co-expression of progranulin and sortilin have worse breast cancer-specific survival.

Figure S4: Patients with high tumor co-expression of progranulin and sortilin have worse breast cancer-specific survival in the ERα positive patient group treated with tamoxifen.

Table S1: Distribution of progranulin scores according to clinicopathological parameters in the cohort.

Table S2: Distribution of sortilin expression according to clinicopathological parameters in the cohort.

Table S3: Cross table.

Table S4: Cox regression analysis on randomized untreated patients with high progranulin tumor tissue expression.

Table S5: Cox regression analysis on all patients.

Table S6: Distribution of co-expression of progranulin and sortilin according to clinicopathological parameters in the cohort.

Table S7: Cox regression analysis on ERα positive patients.

Supplementary Figures and Legends

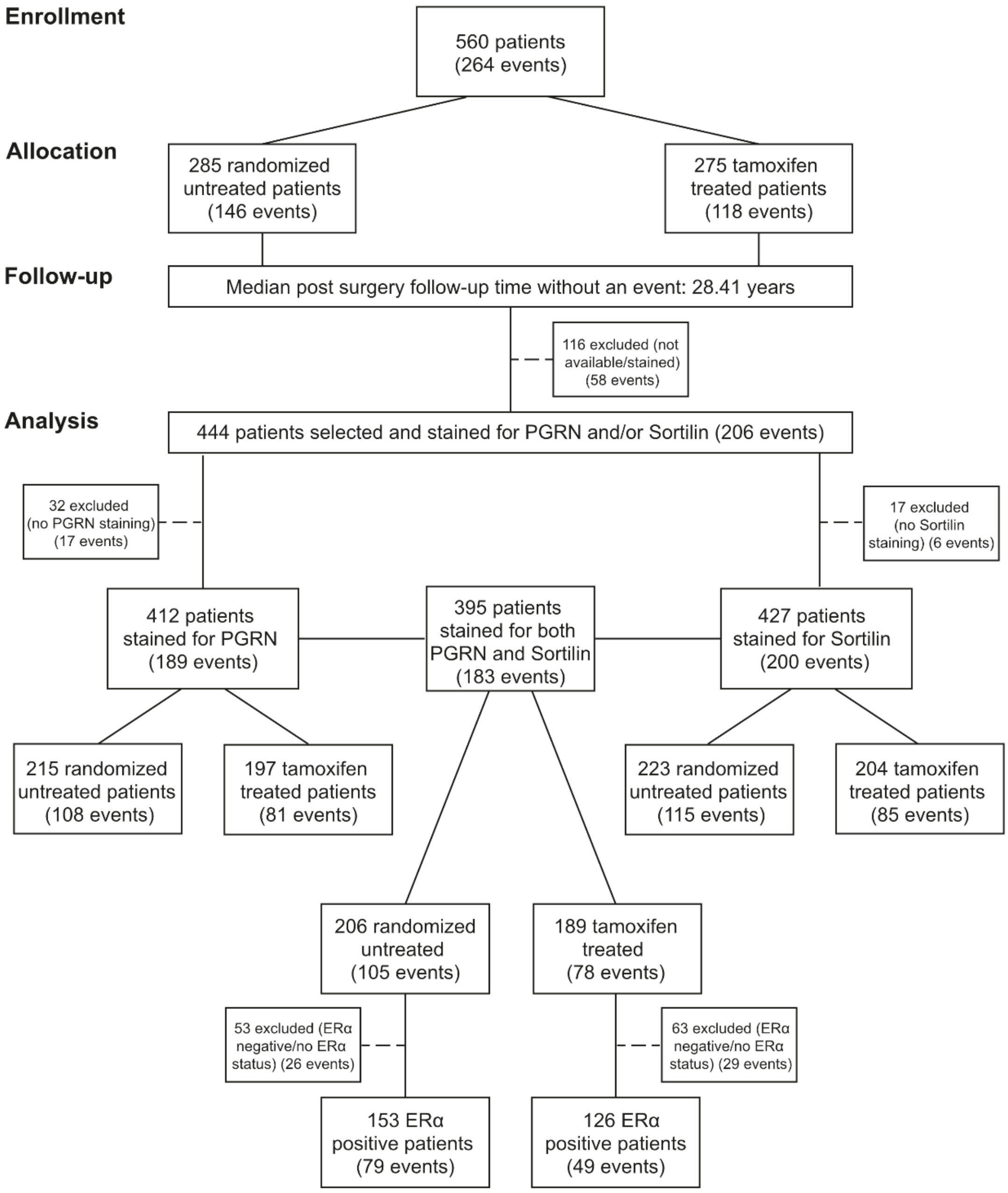

Figure S1: CONSORT diagram for the study. 560 premenopausal breast cancer patients were enrolled in the study and randomized to either two years of tamoxifen treatment or no systemic treatment. TMAs were constructed and stained for progranulin and sortilin expression using IHC and specific antibodies. Patients were followed-up for breast

cancer-specific survival where an event is defined as death from breast cancer. TMA: tissue microarray, IHC: immunohistochemistry, ERα: estrogen receptor alpha PGRN: progranulin.

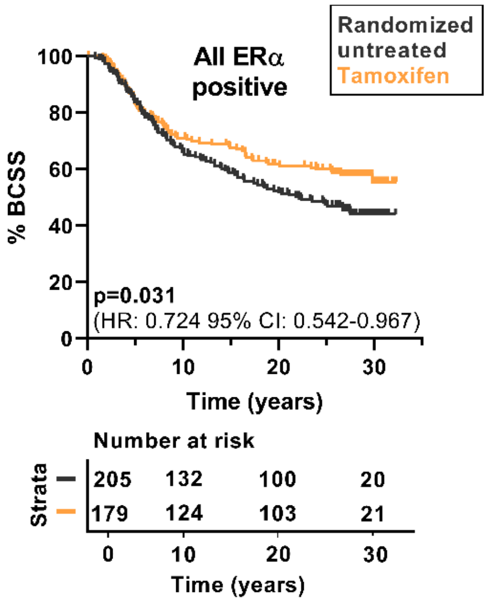

Figure S2: ERα positive patients stratified by treatment arm. Kaplan-Meier estimates showing breast cancer-specific survival in all ERα positive breast cancer patients from the cohort according to treatment arm (n=384). The statistical differences between the curves, as well as HR and 95% CI were estimated by the log-rank test. BCSS: breast cancer-specific survival, HR: hazard ratio, CI: confidence interval.

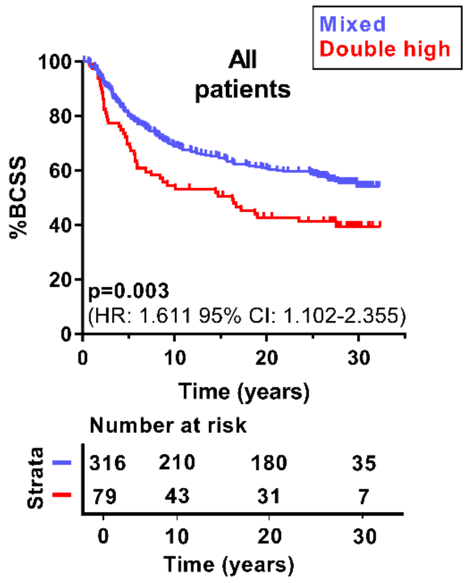

Figure S3: Patients with high tumor co-expression of progranulin and sortilin have worse breast cancer-specific survival. Kaplan-Meier curves illustrating breast cancer-specific survival on combined progranulin and sortilin expression, making a double high group (high progranulin, high sortilin expression) against all other combinations (of low/high progranulin/sortilin expression) in all patients (n=395). The statistical differences between the curves, as well as HR and 95% CI were estimated by the log-rank test. BCSS: breast cancer-specific survival, HR: hazard ratio, CI: confidence interval, PGRN: progranulin, SORT: sortilin.

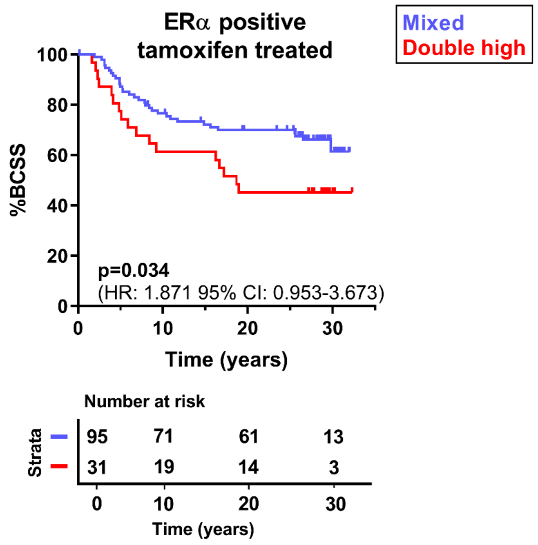

Figure S4: Patients with high tumor co-expression of progranulin and sortilin have worse breast cancer-specific survival in the ERα positive patient group treated with tamoxifen. Kaplan-Meier curves Kaplan-Meier curves illustrating breast cancer-specific survival in ERα positive patients treated with tamoxifen. BCSS in the double high group (high progranulin, high sortilin expression) against all other combinations (mixed group) (*n*=126). The statistical differences between the curves, as well as HR and 95% CI were estimated by the log-rank test. BCSS: breast cancer-specific survival, HR: hazard ratio, CI: confidence interval, PGRN: progranulin, SORT: sortilin.

Supplementary Tables and Legends

Table S1: Distribution of progranulin scores according to clinicopathological parameters in the cohort.

Progranulin expression	1 (n=113)	2 (n=160)	3 (n=123)	4 (n=16)	Value	P-value
**Randomization**						
Untreated	60 (53)	89 (56)	58 (47)	8 (50)		
Tamoxifen	53 (47)	71 (44)	65 (53)	8 (50)	2.075[1]	0.557
Missing: 0						
**Age at diagnosis (year, continuous)**						
Median	44.00	45.00	46.00	43.00		
Range	25-55	31-57	26-57	35-52	6.515[2]	0.089
Missing: 0						
**Tumor size (mm, continuous)**						
Median	23.00	24.00	23.00	27.00		
Range	7-58	2-75	2-50	8-50	2.147[2]	0.542
Missing: 1						
**Tumor histology**						
Ductal	87 (77)	143 (89)	106 (86)	11 (69)		
Lobular	21 (19)	4 (3)	4 (33)	0 (0)		
Medullar	1 (1)	6 (4)	10 (81)	4 (25)	50.897[1]	**<0.001**
Missing: 15						
**Tumor grade**						
Grade 1	22 (20)	24 (15)	5 (4)	1 (6)		
Grade 2	59 (52)	68 (43)	35 (29)	5 (31)		
Grade 3	29 (26)	61 (38)	78 (63)	10 (63)	43.126[1]	**<0.001**
Missing: 15						
**LN status**						
LN positive	82 (73)	108 (68)	89 (72)	10 (63)		
LN negative	31 (27)	50 (31)	34 (28)	6 (37)	4.445[1]	0.617
Missing: 2						
**ERα**						
ERα positive (>10%)	94 (83)	116 (73)	72 (59)	8 (50)		
ERα negative (<10%)	12 (11)	33 (21)	40 (33)	7 (44)	22.220[1]	**<0.001**
Missing: 30						
**PR**						
PR positive (>10%)	64 (57)	87 (54)	52 (42)	3 (19)		
PR negative (<10%)	24 (21)	42 (26)	45 (37)	9 (56)	15.797	**0.001**

Missing: 86						
**HER2**						
HER2 negative	91 (81)	125 (78)	100 (81)	15 (94)		
HER2 positive	14 (12)	26 (16)	18 (15)	0 (0)	3.479[1]	0.323
Missing: 23						
**Ki67**						
<25%	81 (72)	106 (66)	68 (55)	8 (50)		
>25%	15 (13)	35 (22)	41 (33)	7 (44)	15.758[1]	**0.001**
Missing: 51						
**Sortilin expression**						
1	33 (29)	25 (16)	25 (20)	2 (12.5)		
2	26 (23)	47 (29)	26 (21)	3 (19)		
3	35 (31)	66 (41)	54 (44)	9 (56)		
4	11 (10)	17 (11)	14 (11)	2 (12.5)	0.112[3]	**0.026**
Missing: 17						
**HIF1α**						
0-1%	73 (65)	99 (62)	63 (51)	10 (63)		
1-10%	6 (5)	16 (10)	24 (20)	0 (0)		
10-100%	2 (2)	12 (8)	12 (10)	2 (33)	21.453[1]	**0.002**
Missing: 93						

[1] Pearson´s Chi-square

[2] Kruskal-Wallis (continuous variables)

[3] Spearman

(LN: lymph node, ERα: estrogen receptor alpha, PR: progesterone receptor, HER2: human epidermal growth factor receptor 2, Ki67: proliferation marker, HIF1α: hypoxia-inducible factor 1-alpha, percentages in parenthesis. P-values below 0.05 were considered statistically significant and highlighted)

Table S2: Distribution of sortilin expression according to clinicopathological parameters in the cohort.

Sortilin expression	1 (n=92)	2 (n=110)	3 (n=177)	4 (n=48)	Value	P-value
**Randomization**						
Untreated	46 (50)	61 (55)	93 (53)	23 (48)		
Tamoxifen	46 (50)	49 (45)	84 (47)	25 (52)	1.007[1]	0.800
Missing: 0						
**Age at diagnosis (year, continuous)**						
Median	46.00	45.00	44.00	44.50		
Range	25-57	31-56	30-57	26-51	8.289[2]	**0.040**
Missing: 0						
**Tumor size (mm, continuous)**						
Median	25.00	23.50	23.00	25.00		
Range	8-58	2-45	2-50	7-75	0.369[2]	0.946
Missing:1						
**Tumor histology**						
Ductal	64 (70)	92 (84)	160 (90)	43 (90)		
Lobular	15 (16)	8 (7)	7 (4)	2 (4)		
Medullar	8 (9)	8 (7)	5 (3)	0 (0)	24.586[1]	**<0.001**
Missing: 15						
**Tumor grade**						
Grade 1	12 (13)	12 (11)	21 (12)	8 (17)		
Grade 2	38 (41)	39 (35)	77 (44)	22 (46)		
Grade 3	37 (40)	58 (53)	71 (40)	17 (35)	5.665[1]	0.462
Missing: 15						
**LN status**						
LN Positive	69 (75)	78 (71)	122 (69)	30 (63)		
LN Negative	22 (25)	32 (29)	54 (30)	18 (37)	4.364[1]	0.627
Missing: 2						
**ERα**						
ERα positive (>10%)	55 (60)	73 (66)	134 (76)	44 (92)		
ERα negative (<10%)	25 (27)	28 (26)	38 (21)	2 (4)	13.317[1]	**0.004**
Missing: 28						
**PR**						
PR positive (>10%)	35 (38)	45 (41)	103 (58)	36 (75)		
PR negative (<10%)	35 (38)	37 (34)	43 (24)	4 (8)	23.510[1]	**<0.001**
Missing: 89						
**HER2**						
HER2 negative	74 (80)	91 (83)	140 (79)	39 (81)		
HER2 positive	13 (14)	15 (14)	29 (16)	3 (6)	2.727[1]	0.436

Missing: 23						
**Ki67**						
<25%	60 (65)	70 (64)	112 (63)	30 (63)		
>25%	21 (23)	25 (23)	44 (25)	11 (23)	0.184[1]	0.980
Missing: 54						
**Progranulin expression**						
1	33 (36)	26 (24)	35 (20)	11 (23)		
2	25 (27)	47 (43)	66 (37)	17 (35)		
3	25 (27)	26 (24)	54 (31)	14 (29)		
4	2 (2)	3 (3)	9 (5)	2 (4)	0.112[3]	**0.026**
Missing: 32						
**HIF1α**						
0-1%	50 (54)	69 (63)	102 (58)	34 (71)		
1-10%	12 (13)	8 (7)	25 (14)	4 (8)		
10-100%	7 (8)	11 (10)	8 (5)	1 (2)	9.512[1]	0.147
Missing: 96						

[1] Pearson´s Chi-square

[2] Kruskal-Wallis (continuous variables)

[3] Spearman

(LN: lymph node, ERα: estrogen receptor alpha, PR: progesterone receptor, HER2: human epidermal growth factor receptor 2, Ki67: proliferation marker, HIF1α: hypoxia-inducible factor 1-alpha, percentages in parenthesis. P-values below 0.05 were considered statistically significant and highlighted)

Table S3: Cross table. Cross table showing the relationship between progranulin and sortilin expression in the patient cohort.

Distribution of progranulin and sortilin expression					
	**Sortilin low (%)**	**Group**	**Sortilin high (%)**	**Group**	**Total (%)**
**Progranulin low**	131 (33.16)	1	129 (32.66)	2	260
**Progranulin high**	56 (14.18)	3	79 (20.00)	4	135
**Total**	187		208		395 (100)

Table S4: Cox regression analysis on randomized untreated patients with high progranulin tumor tissue expression. Multivariable interaction analysis on breast cancer-specific survival evaluating various prognostic parameters for relative risk estimation for the untreated patient cohort having high tumor expression of progranulin. Multivariable model adjusted for grade, lymph node status, tumor size, age, ERα and HER2 status, in addition to sortilin scoring. HR: hazard ratio, CI: confidence interval, LN: lymph node, ERα: estrogen receptor alpha, HER2: human epidermal growth factor receptor 2.

	Multivariable analysis		
**Variable**	**HR**	**95% CI**	**p**
**Grade**			
I-II	1		
III	1.397	0.531-3.673	0.498
**LN status**			
LN negative	1		
LN positive	3.854	1.666-8.919	**0.002**
**Tumor size**			
Continuous (mm)	1.089	1.037-1.143	**0.001**
**Age**			
Continuous (per year)	0.975	0.920-1.143	0.393
**ERα**			
ERα negative	1		
ERα positive	1.877	0.726-4.854	0.194
**HER2**			
HER2 negative	1		
HER2 positive	0.685	0.238-1.969	0.482
**Sortilin**			
Low	1		
High	3.013	1.219-7.448	**0.017**

Table S5: Cox regression analysis on all patients. Univariate and multivariable interaction analysis on breast cancer-specific survival evaluating various prognostic parameters for relative risk estimation for all patients in the cohort. Multivariable model adjusted for grade, lymph node status, tumor size, age, treatment, ERα and HER2 status, in addition to the progranulin/sortilin scoring combination. HR: hazard ratio, CI: confidence interval, LN: lymph node, ERα: estrogen receptor alpha, HER2: human epidermal growth factor receptor 2.

	Univariate analysis			Multivariable analysis		
**Variable**	**HR**	**95% CI**	**p**	**HR**	**95% CI**	**p**
**Grade**						
I-II	1			1		
III	1.676	1.272-2.210	**<0.001**	1.600	1.109-2.307	**0.012**
**LN status**						
LN negative	1			1		
LN positive	1.520	1.102-2.096	**0.011**	1.617	1.118-2.338	**0.011**
**Tumor size**						
Continuous (mm)	1.014	1.000-1.028	**0.044**	1.021	1.005-1.037	**0.010**
**Age**						
Continuous (per year)	0.982	0.958-1.007	0.159	0.992	0.964-1.021	0.583
**Randomization group**						
Untreated	1			1		
Tamoxifen	0.788	0.598-1.038	0.091	0.710	0.517-0.974	**0.034**
**ERα**						
ERα negative	1			1		
ERα positive	0.749	0.539-1.041	0.085	0.942	0.622-1.427	0.779
**HER2**						
HER2 negative	1			1		
HER2 positive	1.440	0.986-2.103	0.059	1.051	0.672-1.644	0.827
**Progranulin/sortilin combination**						
Mixed	1			1		
Double high	1.612	1.157-2-247	**0.005**	1.723	1.209-2.457	**0.003**

Table S6: Distribution of co-expression of progranulin and sortilin according to clinicopathological parameters in the cohort.

Co-expression Progranulin/sortilin	Double high (n=79)	Other (n=316)	Value	p-value
**Randomization**				
Untreated	37 (47)	169 (53)		
Tamoxifen	42 (53)	147 (47)	1.119[1]	0.290
Missing: 0				
**Age at diagnosis (year, continuous)**				
Median	45.00	45.00		
Range	26-57	25-57	12801.500[2]	0.724
Missing: 0				
**Tumor size (mm, continuous)**				
Median	23.00	24.00		
Range	2-50	2-75	12558.000[2]	0.898
Missing: 1				
**Tumor histology**				
Ductal	73 (92)	263 (83)		
Lobular	0 (0)	26 (8)		
Medullar	3 (4)	17 (5)	7.465[1]	**0.024**
Missing: 13				
**Tumor grade**				
Grade 1	5 (6)	46 (15)		
Grade 2	29 (37)	128 (41)		
Grade 3	42 (13)	131 (42)	5.532[1]	0.063
Missing: 14				
**LN status**				
LN Positive	55 (70)	223 (71)		
LN Negative	24 (30)	91 (28)	0.562[1]	0.755
Missing: 2				
**ERα**				
ERα positive (>10%)	55 (70)	224 (71)		
ERα negative (<10%)	21 (27)	68 (22)	0.621[1]	0.431
Missing: 27				
**PR**				
PR positive (>10%)	42 (53)	153 (48)		
PR negative (<10%)	23 (29)	92 (29)	0.103[1]	0.748
Missing: 85				
**HER2**				
HER2 negative	66 (84)	251 (79)		
HER2 positive	10 (13)	48 (15)	0.389[1]	0.533

Missing: 20				
**Ki67**				
<25%	46 (58)	207 (66)		
>25%	26 (33)	70 (22)	3.368[1]	0.066
Missing: 46				
**HIF1α**				
0-1%	44 (56)	194 (61)		
1-10%	13 (17)	32 (10)		
10-100%	5 (6)	21 (7)	2.565[1]	0.277
Missing: 86				

[1] Pearson´s Chi-square

[2] Mann-Whitney U Test (continuous variables)

[3] Spearman

(LN: lymph node, ERα: estrogen receptor alpha, PR: progesterone receptor, HER2: human epidermal growth factor receptor 2, Ki67: proliferation marker, HIF1α: hypoxia-inducible factor 1-alpha, percentages in parenthesis. P-values below 0.05 were considered statistically significant and highlited)

Table S7: Cox regression analysis on ERα positive patients. Multivariable regression analysis on breast cancer-specific survival evaluating various prognostic parameters for relative risk estimation for the ERα positive patient cohort. Multivariable model adjusted for grade, lymph node status, tumor size, age, treatment, HER2 status, as well as the progranulin/sortilin scoring combination. HR: hazard ratio, CI: confidence interval, LN: lymph node, ERα: estrogen receptor alpha, HER2: human epidermal growth factor receptor 2.

	**Multivariable analysis**		
**Variable**	**HR**	**95% CI**	**p**
**Grade**			
I-II	1		
III	1.612	1.086-2.394	**0.018**
**LN status**			
LN negative	1		
LN positive	1.453	0.940-2.244	0.093
**Tumor size**			
Continuous (mm)	0.999	0.980-1.019	0.940
**Age**			
Continuous (per year)	0.971	0.939-1.005	0.093
**Randomization group**			
Untreated	1		
Tamoxifen	0.628	0.431-0.915	**0.015**
HER2			
HER2 negative	1		
HER2 positive	1.716	1.027-2.867	**0.039**
**Progranulin/sortilin combination**			
Mixed	1		
Double high	1.980	1.308-2.996	**0.001**
