# Supplementary material for: Tumor co-expression of progranulin and sortilin as a prognostic biomarker in breast cancer
Source: BMC Cancer. 2021 Feb 22;21:185. doi: 10.1186/s12885-021-07854-0 (PMC7898426; doi:10.1186/s12885-021-07854-0)
Supplement: Supplementary file 6 — Additional file 6. Sortilin tumor expression on its own shows no difference in survival. Kaplan-Meier curves illustrating breast cancer-specific survival according to high or low sortilin expression. [file 12885_2021_7854_MOESM6_ESM.pdf]

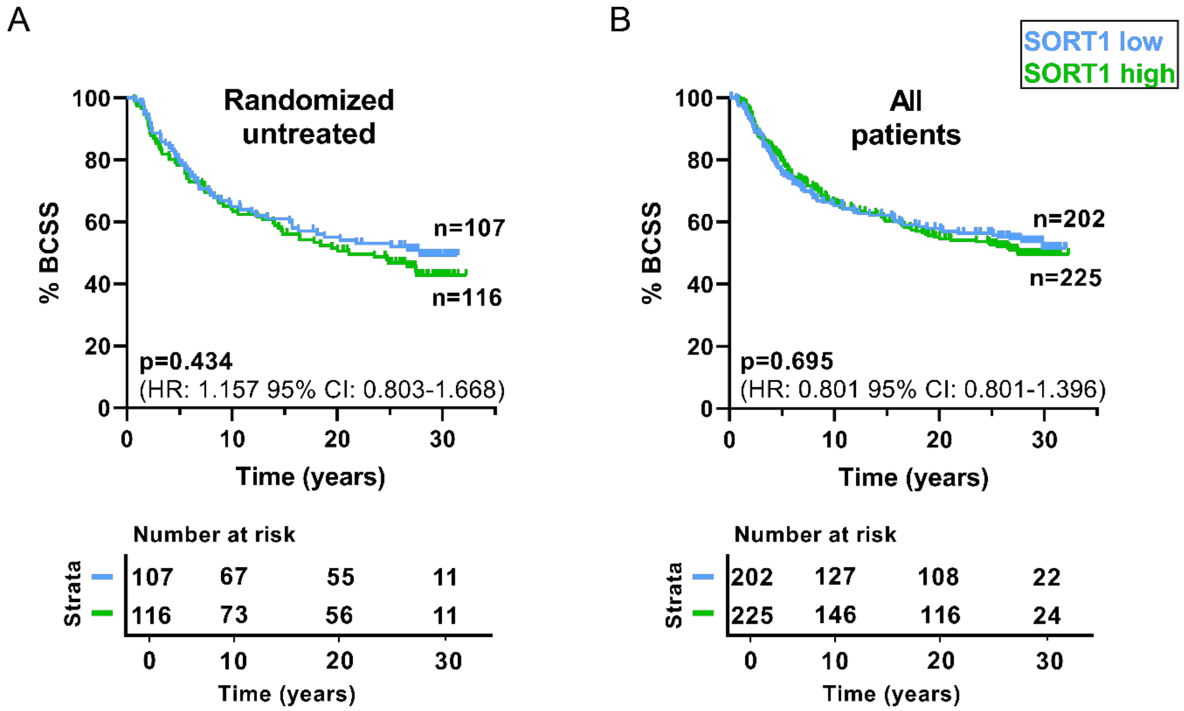

Additional file 6: Sortilin tumor expression on its own shows no difference in survival. Kaplan-Meier curves illustrating breast cancer-specific survival according to high or low sortilin expression, in randomized untreated patients only (A) (n=223) or all patients (n=427) (B). The statistical differences between the curves, as well as HR and 95% CI were estimated by the log-rank test. BCSS: breast cancer-specific survival, HR: hazard ratio, CI: confidence interval, SORT1: sortilin.
